# Photon-Counting Detector CT-Based Virtual Monoenergetic Imaging With Metal Artifact Reduction for Endodontic Diagnostics

**DOI:** 10.1016/j.identj.2026.109547

**Published:** 2026-04-23

**Authors:** Adib Al-Haj Husain, Selinay Dogan, Victor Mergen, Tristan T. Demmert, Hatem Alkadhi, Thomas Flohr, Harald Essig, Bernd Stadlinger, Egon Burian

**Affiliations:** a Department of Cranio-Maxillofacial and Oral Surgery, University Hospital Zurich, University of Zurich, Zurich, Switzerland; b Clinic of Cranio-Maxillofacial and Oral Surgery, Center of Dental Medicine, University of Zurich, Zurich, Switzerland; c Department of Cranio-Maxillofacial Surgery, GROW School for Oncology and Reproduction, Maastricht University Medical Centre, Maastricht, The Netherlands; d Diagnostic and Interventional Radiology, University Hospital Zurich, University of Zurich, Zurich, Switzerland; e Department of Radiology and Nuclear Medicine, Maastricht University Medical Centre, Maastricht, the Netherlands

**Keywords:** Endodontics, Photon-counting detector computed tomography, Virtual monoenergetic imaging, Metal artifact reduction, Dental imaging

## Abstract

**Introduction:**

This *ex vivo* study aimed to determine the optimal energy level for virtual monoenergetic imaging (VMI) using photon-counting detector computed tomography (PCD-CT) and to evaluate the effectiveness of iterative metal artifact reduction (iMAR) in assessing simulated endodontic challenges and complications.

**Methods:**

Sixteen extracted third molars were simulated with one of eight distinct endodontic diagnostic challenges and imaged using PCD-CT at radiation doses equivalent to standard-dose cone-beam CT. VMIs were reconstructed from 70-190 keV at 10 keV increments, both with and without iMAR. Diagnostic accuracy, depiction quality of endodontic challenges, artifact severity, and visualization of key endodontic anatomical structures were independently assessed by 2 observers using a 5-point visual analogue scale (1 = least favourable, 5 = most favourable). Descriptive statistics were calculated, and inter-reader agreement was analysed using Krippendorff’s alpha coefficient.

**Results:**

VMIs achieved excellent diagnostic accuracy (97%) and high-quality visualization of endodontic challenges (median: 5, IQRs: 4-5 or 4.25-5; α = 0.63-1.00) across the entire reconstructed energy spectrum (70-190 keV), with minimal artifacts, particularly at ≥ 110 keV (α = 1.0). Task-specific analysis demonstrated optimal visualization of caries at 70-80 keV and fractured files at 70-100 keV. For other pathologies, VMI at ≥100 keV effectively reduced artifacts without compromising anatomical detail. IMAR did not improve image quality and consistently reduced diagnostic performance.

**Conclusions:**

VMI from PCD-CT provides high-quality imaging with minimal artifacts, well-suited for indication-specific endodontic diagnostics.

**Clinical Relevance:**

PCD-CT supports novel, indication-specific workflows in endodontic imaging, potentially enhancing diagnostic precision and long-term treatment follow-up.

## Introduction

Beyond clinical assessment, imaging-based decision-making has become a crucial component of endodontic diagnostics, supporting initial assessment, treatment planning, and follow-up.^1^ Furthermore, repeated imaging during treatment is valuable for managing the technical complexity of intraoperative procedures, particularly during key phases such as root canal instrumentation, chemical and mechanical debridement, and canal sealing, thereby ensuring procedural accuracy and supporting favourable long-term outcomes.^2^

Conventional intraoral periapical radiographs and cone-beam computed tomography (CBCT) are the most commonly used imaging techniques in clinical practice due to their high accessibility, cost-effectiveness, relatively low radiation exposure, and widespread familiarity among dental professionals.^1^ In clinical practice, CBCT-based 3-dimensional imaging offers superior diagnostic performance, particularly in complex endodontic conditions, when compared to 2-dimensional periapical radiographs; however, it is associated with higher radiation exposure.^3^^,^^4^ Therefore, current clinical-decision-making analysis supports its use primarily as an adjunct to treatment planning rather than as a first-line diagnostic tool, with the majority of endodontic CBCT examinations being performed in previously root-filled teeth.^5^ With each modality having inherent limitations, periapical radiographs often exhibit diagnostic inaccuracies due to the overlapping of pathoanatomical structures. In contrast, CBCT imaging can be limited by low contrast resolution and artifacts arising from various endodontic materials, such as obturators, cements, and metallic posts.^6^^,^^7^

Photon-counting detector computed tomography (PCD-CT) has recently been adopted in clinical practice, utilizing energy-resolving cadmium telluride semiconductors that directly convert incoming X-ray photons into electrical signals. This technology offers superior radiation dose efficiency,^8^ minimizes artifacts,^9^ provides high spatial resolution^10^ comparable to that of CBCT, and has inherent spectral capabilities, thereby opening promising new opportunities in endodontic imaging.^11^^,^^12^

Previous ex vivo studies have demonstrated comparable or superior diagnostic performance of PCD-CT relative to CBCT at matched radiation-dose levels.^13^ Across various challenging endodontic diagnostic tasks, including lesion detection, working length assessment, and identification of vertical root fractures or small accessory canals in previously root-filled teeth, PCD-CT has shown high diagnostic accuracy, even at reduced dose settings.^11^^,^^14^, ^15^, ^16^

However, beyond general modality comparisons, the specific contribution of virtual monoenergetic images with metal artifact algorithms that leverage the inherent spectral information of PCD-CT^17^ in endodontic imaging has not yet been systematically investigated, despite their demonstrated clinical value across various other medical disciplines.

Thus, this *ex vivo* study aimed to determine the optimal energy level for VMI using PCD-CT and to evaluate the effectiveness of iMAR in assessing simulated endodontic challenges and complications.

## Materials and methods

### Study design and ethics

A total of 16 extracted human mandibular third molars, checked for the absence of extraction-related damage, were collected from the clinical routine of the Department of Cranio-Maxillofacial and Oral Surgery to simulate endodontic lesions and complications relevant to endodontic procedures.

Eight distinct endodontic diagnostic scenarios and complications, including carious lesions, root canal fillings, fractured instruments, root perforations, and external root resorption, were simulated: Teeth 1 and 2 remained unmodified and served as baseline controls. Teeth 3 and 4 were selected based on the presence of carious lesions. Teeth 5 and 6 underwent root canal instrumentation and were temporarily filled with Cavit (3M Espe). In teeth 7 and 8, root canals were obturated using gutta-percha (FKG Dentaire, La Chaux-de-Fonds, Switzerland) in combination with a root canal sealer. Teeth 9 and 10 were manipulated to simulate a fractured Hedstrom file in the apical third (FKG Dentaire). Tooth 11 had a simulated root perforation in the distal root, while tooth 12 had the same perforation followed by full endodontic therapy. Tooth 13 had a cast post placed with a root perforation, whereas tooth 14 also had the same simulated lesion, followed by full endodontic treatment. Teeth 15 and 16 were prepared to replicate external root resorption.

Endodontic treatment was performed through the following protocol: After creating an access cavity, patency was confirmed using a size 10 K-file (Dentsply Sirona Endodontic). The working length was established at 1 mm short of the apical foramen. Canal shaping was accomplished using the ProTaper Profile Orifice Shapers System (Dentsply Sirona). Irrigation was carried out using 5 mL of 2.5% sodium hypochlorite. Canals were then obturated using the lateral condensation technique with gutta-percha and AH Plus resin sealer (Dentsply). Where applicable, titanium posts (Titanium (Mooser) Cendres + Métaux SA) were inserted per manufacturer guidelines. External root resorption was artificially created using a 1.1 mm round diamond bur (FG 4200, Intensive).

All specimens underwent PCD-CT imaging at a radiation dose equivalent to that of a standard-dose reference CBCT scan. The imaging sessions were conducted and monitored by trained research staff from the Departments of Radiology and Cranio-Maxillofacial and Oral Surgery of the University of Zurich.

Ethical approval was not required, as confirmed by the Cantonal Ethics Commitee of Zurich (Switzerland). All procedures adhered to the Declaration of Helsinki and its latest amendments regarding medical research.

### Imaging data acquisition

The specimens were scanned using a first-generation dual-source PCD-CT system (NAEOTOM Alpha; Siemens Healthineers AG) equipped with 2 cadmium telluride detectors. Image acquisition was performed with a detector collimation of 120 × 0.2 mm, a tube voltage of 140 kV, and a pitch of 0.85. Radiation dose parameters for PCD-CT scans were matched to the previously scanned reference using the manufacturer’s recommended standard-dose protocol for CBCT (NewTom 7G; QR Systems). The volume CT dose index (CTDI_vol_) was set to 2.82 mGy, resulting in a dose-length product (DLP) of 36.9 mGy • cm and an effective dose of 74 μSv, based on a conversion factor of 0.002 mSv/mGy/cm.^18^

VMIs were reconstructed from 70 to 190 keV at 10 keV increments, using the Hr76 kernel and Quantum Iterative Reconstruction (QIR) at level 3. For reconstructions using iMAR algorithms, VMIs were reconstructed using the Hr56 kernel (the sharpest kernel available in combination with iMAR) using QIR level 3. All reconstructions were performed with a slice thickness and increment of 0.4 mm, and applying a matrix size of 1024 × 1024 pixels.

### Image analysis

All reconstructions were analysed for the following parameters: lesion classification, depiction of the simulated endodontic condition, and artifact susceptibility were rated using 5-point analogue visual scales by 2 observers with different levels of expertise and specialization: Observer A (A.A.H.) is a resident in oral and maxillofacial surgery with 4 years of experience, while Observer B (S.D.) is a general dentist with 1 year of clinical experience. A calibration session, led by one of the principal investigators, was conducted to ensure that all readers had a consistent understanding of the evaluation criteria. Evaluations were conducted in a randomized order, with observers blinded to each other's assessments and the reconstruction parameters.

The simulated endodontic lesions and complications were categorized using a 3-point diagnostic accuracy scale: 0, task or complication not identified; 1, task or complication identified with an incorrect diagnosis; and 2, task or complication identified with a correct diagnosis.

The depiction quality of endodontic tasks was evaluated using a 5-point analogue visual scale: 5, excellent depiction, with clear visualization and high diagnostic confidence including fine anatomical detail; 4, good depiction, clearly visible with good diagnostic confidence; 3, moderate depiction, identifiable but with some ambiguity; 2, suboptimal depiction, barely visible with low diagnostic confidence; 1, non-diagnostic depiction, not discernible.

The presence and severity of image artifacts were assessed using a 5-point scale: 5, no artifacts present; 4, minimal streak artifacts with no impact on interpretation; 3, moderate streak artifacts with impact on interpretation; 2, significant artifact, substantial interference with interpretation; 1, non-diagnostic due to severe artifacts.

The visualization of key endodontic anatomical structures (enamel, dentin, pulp chamber, root canals, and apical foramen) was graded as follows: 5, fine details visible, fully diagnostic; 4, small details visible, diagnostically adequate; 3, only broad details visible, limited diagnostic value; 2, major structures poorly visible, non-diagnostic; 1, no discernible structures.

### Statistical analysis

All statistical analyses were performed using IBM SPSS Statistics software (version 29.0.2.0, IBM), with a significance level set at α = 0.05. Descriptive statistics were used to summarize qualitative data, including the calculation of medians and interquartile ranges (IQR). Diagnostic classification accuracy was expressed as a percentage. In addition to overall diagnostic accuracy, sensitivity, specificity, and 95% confidence intervals (CIs) were calculated for the diagnostic classification of simulated endodontic tasks. Krippendorff’s alpha coefficient was used to evaluate inter-observer agreement, where α = 1 signifies perfect reliability, α = 0 reflects reliability equivalent to chance, and α < 0 implies systematic disagreement.^19^ To further evaluate the effect of iMAR, the task-specific optimal VMI energy level reconstructed without iMAR was statistically compared with the corresponding reconstruction at the same energy level including iMAR. Paired comparisons were performed using the Wilcoxon signed-rank test.

## Results

A total of 14 PCD-CT reconstructions, including 13 VMI levels (70-190 keV) and the task-specific optimal VMI level with iMAR, were analysed across eight different endodontic diagnostic tasks, resulting in 224 evaluations per observer.

Diagnostic performance, based on the classification outcomes, yielded a sensitivity of 96.8% (95% CI: 92.4-99.1%) and a specificity of 98.2% (95% CI: 94.7-99.6%).

**Fig. 1:**
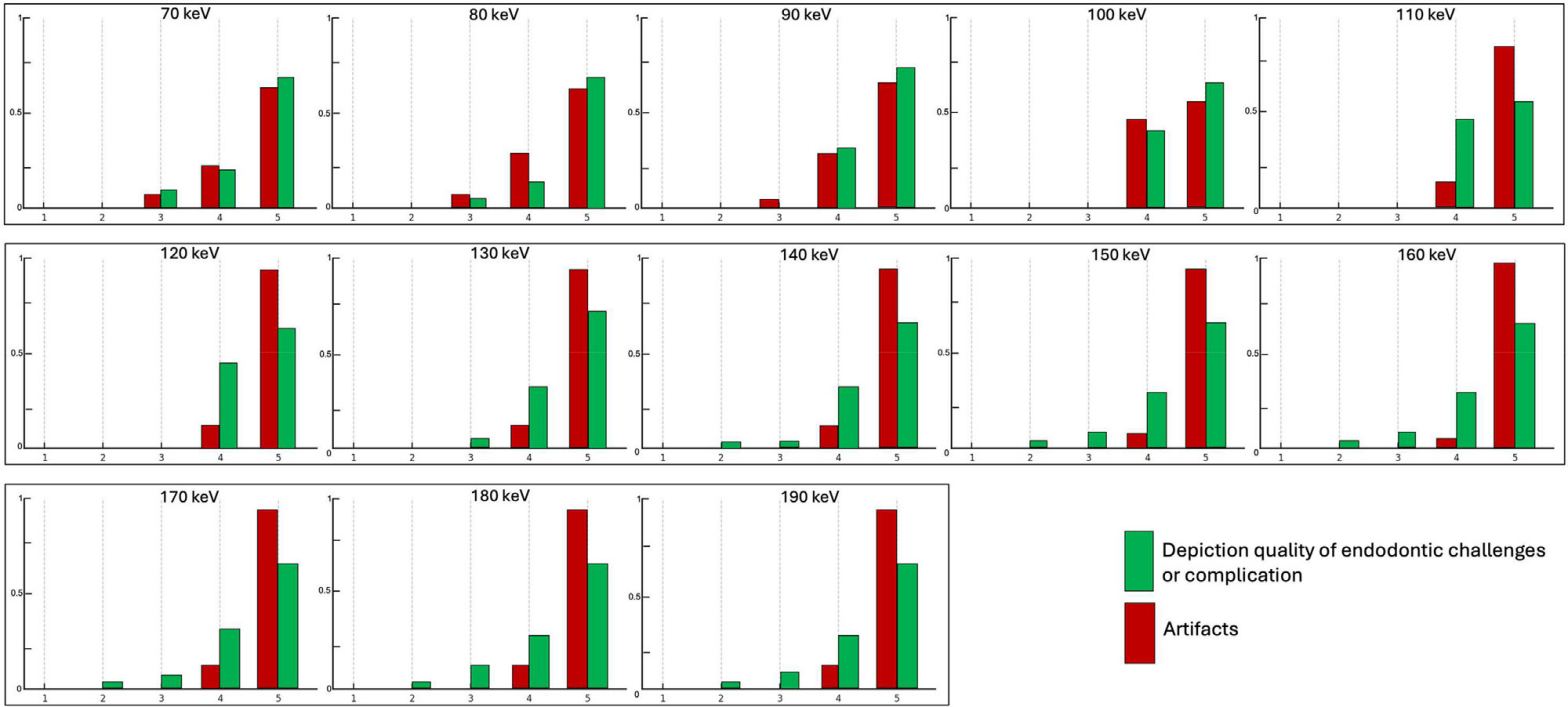
Frequency distribution of visual grading scores (5 = most favourable, 1 = least favourable) from qualitative assessments of image quality, and artifact susceptibility in PCD-CT-based VMI reconstructions (70 to 190 keV, 10 keV increments).

Protocol-specific analysis demonstrated that the depiction quality of endodontic challenges was consistently high across all VMI levels, with median scores of 5 and interquartile ranges of 4-5 or 4.25-5. Interobserver agreement for diagnostic assessment ranged from 0.63 to 1.00, indicating moderate to perfect reliability. Artifacts were minimal to absent across all VMI levels for all pathologies, with predominantly artifact-free visualization achieved at VMI levels of 110 keV or higher. Inter-observer agreement for artifact evaluation was consistently high (α = 0.88-1.00). Detailed results and frequency distributions are provided in Table 1 and Figure 1**.**

**Table 1:** Depiction quality of simulated endodontic challenges and complications, as well as artifact susceptibility, were qualitatively assessed by 2 independent observers using a 5-point scale (5 = most favourable, 1 = least favourable) across 13 VMI-level PCD-CT reconstructions (70-190 keV, 10 keV increments). Results are reported as medians with interquartile ranges in parentheses, and inter-observer agreement is expressed using Krippendorff’s α

	Imaging Protocol	Observer A	Observer B	Inter-observer agreement
Depiction quality of endodontic challenge	70 keV	5 (4-5)	5 (4-5)	0.63
	80 keV	5 (4.25-5)	5 (4-5)	0.78
	90 keV	5 (4-5)	5 (4-5)	0.71
	100 keV	5 (4-5)	5 (4-5)	0.88
	110 keV	5 (4-5)	5 (4-5)	0.88
	120 keV	5 (4-5)	5 (4-5)	0.76
	130 keV	5 (4-5)	5 (4-5)	0.76
	140 keV	5 (4-5)	5 (4-5)	0.78
	150 keV	5 (4-5)	5 (4-5)	0.88
	160 keV	5 (4-5)	5 (4-5)	0.88
	170 keV	5 (4-5)	5 (4-5)	0.88
	180 keV	5 (4-5)	5 (4-5)	0.88
	190 keV	5 (4-5)	5 (4-5)	1
Artifacts	70 keV	5 (4-5)	5 (4-5)	1
	80 keV	5 (4-5)	5 (4-5)	1
	90 keV	4 (4-5)	4.5 (4-5)	0.89
	100 keV	4.5 (4-5)	4 (4-5)	0.88
	110 keV	5 (5-5)	5 (5-5)	1
	120 keV	5 (5-5)	5 (5-5)	1
	130 keV	5 (5-5)	5 (5-5)	1
	140 keV	5 (5-5)	5 (5-5)	1
	150 keV	5 (5-5)	5 (5-5)	1
	160 keV	5 (5-5)	5 (5-5)	1
	170 keV	5 (5-5)	5 (5-5)	1
	180 keV	5 (5-5)	5 (5-5)	1
	190 keV	5 (5-5)	5 (5-5)	1

Interpretation of Krippendorff’s α: 1 = perfect reliability, 0 = no reliability beyond chance, <0 = systematic disagreement

**Fig. 2:**
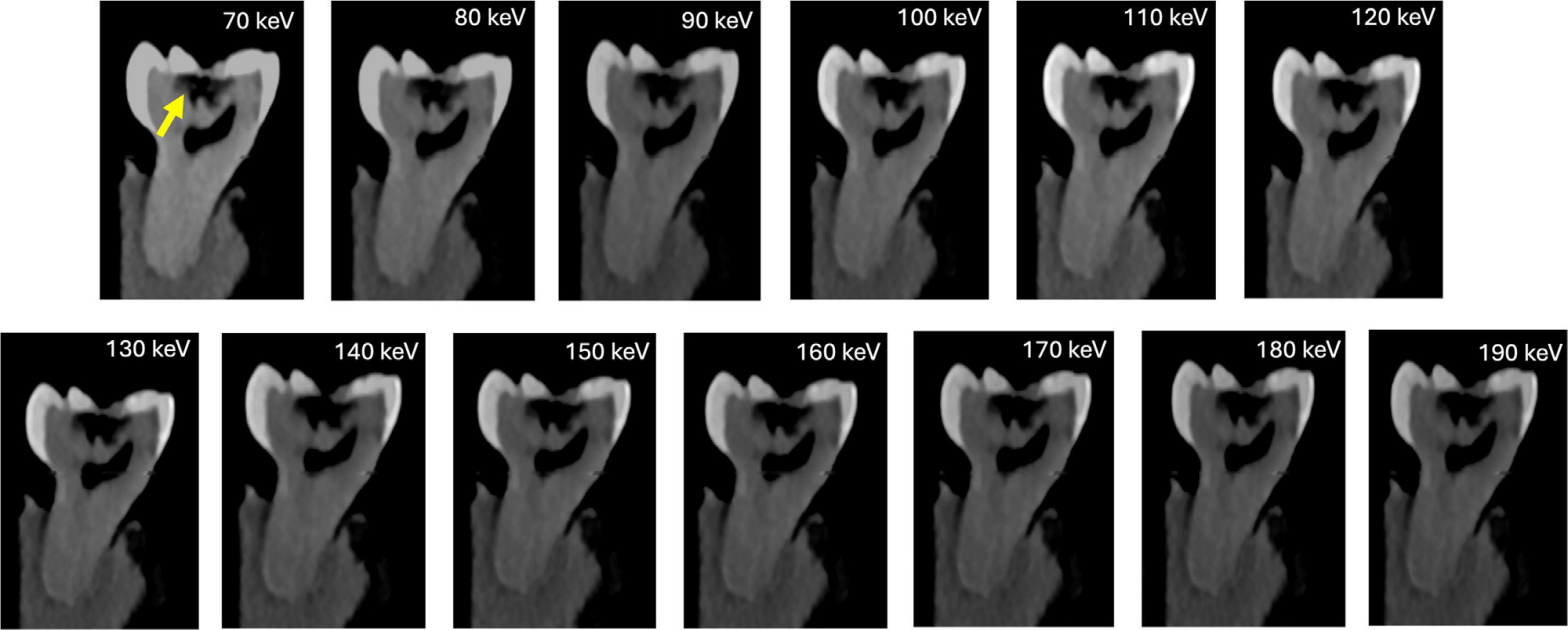
Carious lesion (arrow) depiction across PCD-CT VMI reconstructions (70-190 keV). Lesions showed clear and confident visualization at low VMI levels (70–80 keV), with a slight drop at higher levels (≥100 keV). Artifacts were minimal or absent across all VMI levels.

**Fig. 3:**
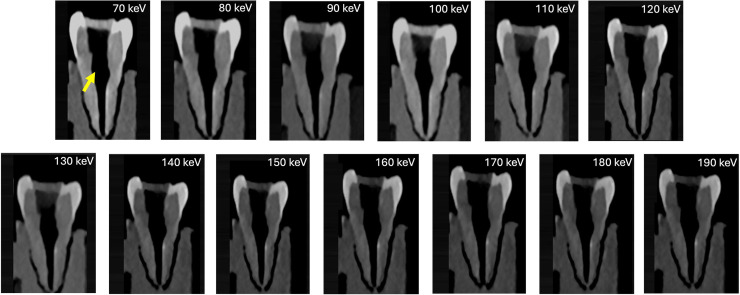
Depiction of instrumented canals filled with Cavit, showing excellent visualization across all keV levels, with no associated artifacts.

**Fig. 4:**
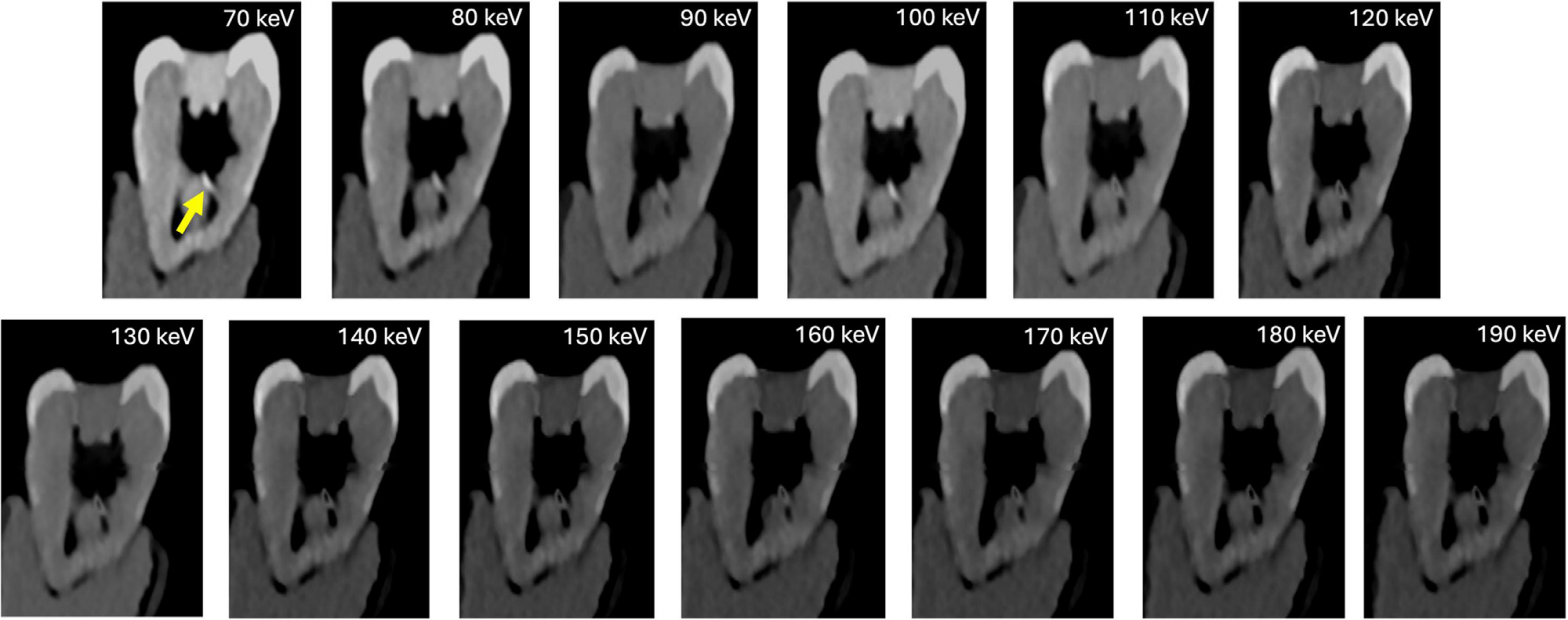
Fractured endodontic Hedstrom file (arrow) depicted across all VMI levels (70-190 keV), reliably identified at 70 to 100 keV, with depiction scores progressively decreasing at higher VMI levels.

**Fig. 5:**
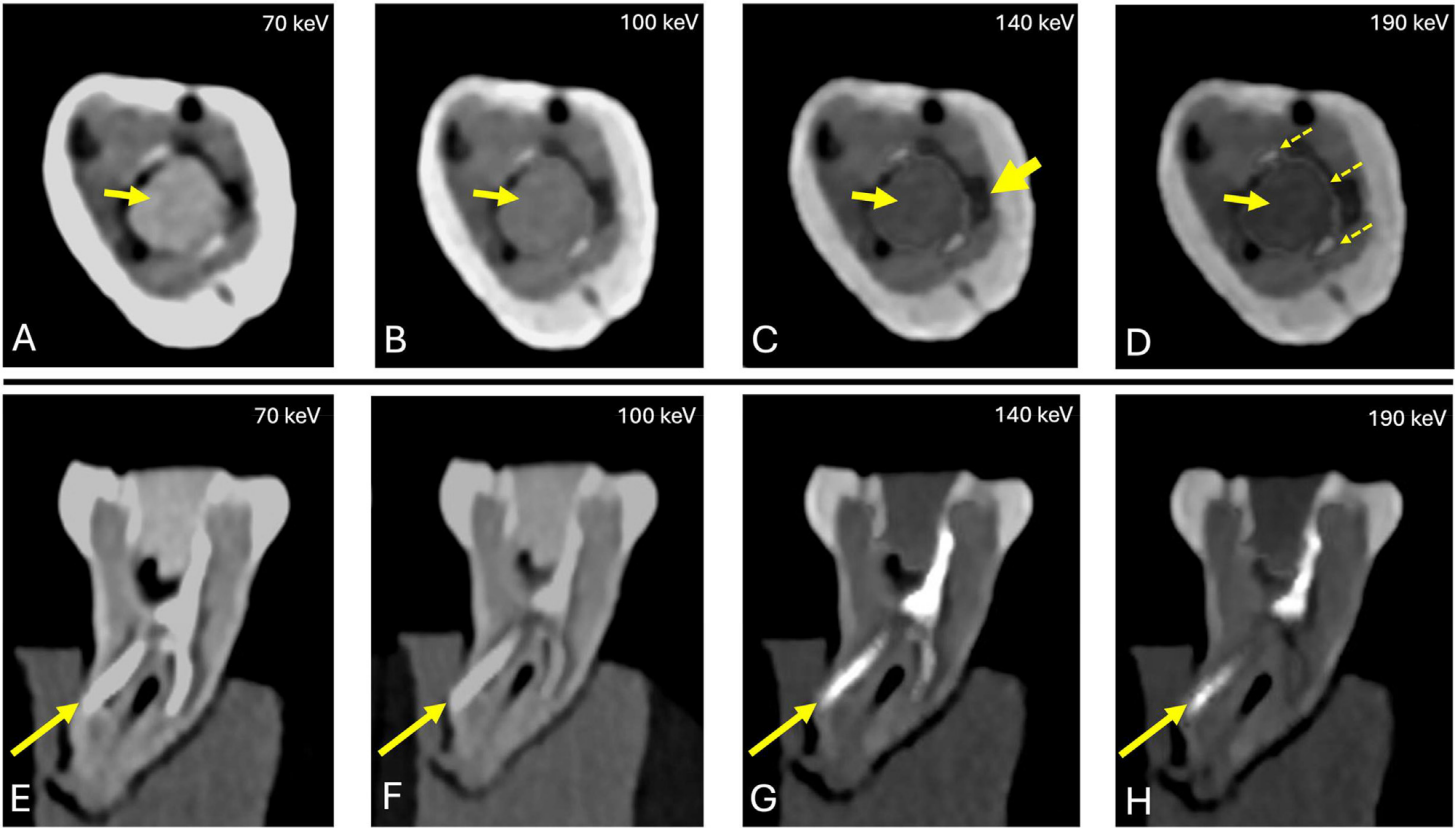
Gutta-percha (left arrow) with root canal sealer showed reduced depiction quality at lower keV levels (A-B), improving to excellent visualization at higher levels (140 and 190 keV). Image (C) highlights a gap between the gutta-percha and the tooth (thick arrow), while in (D) the thin pointed arrows indicate the thin sealer layer surrounding the gutta-percha at 190 keV. Panels (E-H) illustrate root perforations with root canal fillings.

Task-specific sub-analysis demonstrated robust and reliable visualization of all endodontic pathologies across the evaluated VMI range. Carious lesions were consistently depicted with high diagnostic confidence and patho-anatomical detail at lower VMI levels (70–80 keV) (median = 5; IQR: 5-5), with a slight decline at higher VMI levels (≥100 keV) (median: 4, IQR: 4–4). Artifacts were minimal to absent across all VMI levels (median = 5; IQR: 5–5) (Figure 2). Instrumented canals filled with Cavit demonstrated excellent depiction across all keV levels (median = 5, IQR: 5-5), with no associated artifacts (Figure 3). Gutta-percha with root canal sealer exhibited reduced depiction quality at lower keV levels (median: 3.5-4), accompanied by concurrent artifact presence (median: 3.5-4). At higher VMI levels (≥120 keV), depiction quality improved to 5 (IQR: 5-5) with minimal to absent artifacts. Fractured endodontic files were reliably identified at 70 to 100 keV, with depiction scores decreasing progressively with increasing VMI levels (median: 3-3.5). Despite minor artifacts at lower VMI levels (median: 4-5), these did not compromise interpretability (Figure 4**)**. Root perforations, external resorptions, and complex cases involving posts with perforations (Figure 5**)** maintained high depiction scores across all reconstructions, with minor artifacts observed primarily at lower VMI levels (Table 2).

**Table 2:** Task-specific analysis of depiction quality across eight endodnotic diagnostic tasks, rated on a 5-point scale (5 = excellent, 1 = non-diagnostic) across PCD-CT-based VMI reconstructions (70-190 keV)

		70 keV	80 keV	90 keV	100 keV	110 keV	120 keV	130 keV	140 keV	150 keV	160 keV	170 keV	180 keV	190 keV
Caries	Depiction	5 (5-5)	5 (5-5)	5 (4.25-5)	4 (4-4)	4 (4-4)	4 (4-4)	4 (4-4)	4 (4-4)	4 (4-4)	4 (4-4)	4 (4-4)	4 (4-4)	4 (4-4)
	Artifacts	5 (5-5)	5 (5-5)	5 (5-5)	5 (5-5)	5 (5-5)	5 (5-5)	5 (5-5)	5 (5-5)	5 (5-5)	5 (5-5)	5 (5-5)	5 (5-5)	5 (5-5)
Instrumented root canal with Cavit	Depiction	5 (5-5)	5 (5-5)	5 (5-5)	5 (4.25-5)	5 (5-5)	5 (5-5)	5 (5-5)	5 (5-5)	5 (5-5)	5 (5-5)	5 (5-5)	5 (5-5)	5 (5-5)
	Artifacts	5 (5-5)	5 (5-5)	5 (5-5)	5 (5-5)	5 (5-5)	5 (5-5)	5 (5-5)	5 (5-5)	5 (5-5)	5 (5-5)	5 (5-5)	5 (5-5)	5 (5-5)
Gutta percha obturation with sealer	Depiction	3.5 (3-4)	3.5 (3-4)	4 (4-4)	4 (4-4)	5 (4.25-5)	5 (5-5)	5 (5-5)	5 (5-5)	5 (5-5)	5 (5-5)	5 (5-5)	5 (5-5)	5 (5-5)
	Artifacts	3.5 (3-4)	3.5 (3-4)	4 (3.25-4)	4 (4-4)	4 (4-4.75)	5 (5-5)	5 (5-5)	5 (5-5)	5 (5-5)	5 (5-5)	5 (5-5)	5 (5-5)	5 (5-5)
Fractured endodontic file	Depiction	5 (5-5)	5 (5-5)	5 (4.25-5)	5 (5-5)	4 (4-4)	4 (4-4)	4 (3.25-4)	3.5 (3-4)	3 (3-3)	3 (3-3.75)	3 (3-3.75)	3 (3-3.75)	3.5 (3-4)
	Artifacts	4 (4-4.25)	4 (4-4)	4 (4-4.75)	5 (4.25-5)	5 (5-5)	5 (5-5)	5 (5-5)	5 (5-5)	5 (5-5)	5 (5-5)	5 (5-5)	5 (5-5)	5 (5-5)
Root perforation	Depiction	5 (4.25-5)	5 (4.25-5)	5 (4.25-5)	5 (4.25-5)	5 (4.25-5)	5 (4.25-5)	5 (4.25-5)	5 (4.25-5)	5 (4.25-5)	5 (4.25-5)	5 (4.25-5)	5 (4.25-5)	5 (4.25-5)
	Artifacts	4.5 (4-5)	4.5 (4-5)	4.5 (4-5)	4.5 (4-5)	4.5 (4-5)	4.5 (4-5)	4.5 (4-5)	4.5 (4-5)	4.5 (4-5)	4.5 (4-5)	4.5 (4-5)	4.5 (4-5)	4.5 (4-5)
Post placement with root perforation	Depiction	5 (4.25-5)	5 (5-5)	5 (5-5)	5 (5-5)	5 (5-5)	5 (5-5)	5 (5-5)	5 (5-5)	5 (5-5)	5 (5-5)	5 (5-5)	5 (5-5)	5 (5-5)
	Artifacts	3.5 (3-4)	4 (4-4)	4 (4-4)	4 (4-4)	4 (4-4)	5 (5-5)	5 (5-5)	5 (5-5)	5 (5-5)	5 (5-5)	5 (5-5)	5 (5-5)	5 (5-5)
External root resorption	Depiction	5 (5-5)	5 (5-5)	5 (5-5)	5 (5-5)	5 (5-5)	5 (5-5)	5 (5-5)	5 (5-5)	5 (5-5)	5 (5-5)	5 (5-5)	5 (5-5)	5 (5-5)
	Artifacts	5 (5-5)	5 (5-5)	5 (5-5)	5 (5-5)	5 (5-5)	5 (5-5)	5 (5-5)	5 (5-5)	5 (5-5)	5 (5-5)	5 (5-5)	5 (5-5)	5 (5-5)

Key anatomical structures (enamel, dentin, pulp chamber, root canals, apical foramen) were perfectly visualized (median = 5; IQR: 5-5) at ≥ 100 keV, while enamel and dentin scored slightly lower (median = 4; IQR: 4-4) at 70-80 keV; inter-observer agreement was perfect (α = 1.00) (Table 3).

**Table 3:** Qualitative assessment of key anatomical structures (enamel, dentin, pulp chamber, root canals, apical foramen) relevant to endodontic diagnostic interpretability, rated by 2 independent readers using a 5-point scale (5 = most favourable, 1 = least favourable)

	70 keV	80 keV	90 keV	100 keV	110 keV	120 keV	130 keV	140 keV	150 keV	160 keV	170 keV	180 keV	190 keV
Enamel	4 (4-4)	4 (4-4)	4 (4-4)	5 (4.25-5)	5 (5-5)	5 (5-5)	5 (5-5)	5 (5-5)	5 (5-5)	5 (5-5)	5 (5-5)	5 (5-5)	5 (5-5)
Dentin	4 (4-4)	4 (4-4)	4 (4-4)	5 (5-5)	5 (5-5)	5 (5-5)	5 (5-5)	5 (5-5)	5 (5-5)	5 (5-5)	5 (5-5)	5 (5-5)	5 (5-5)
Pulp Chamber	5 (5-5)	5 (5-5)	5 (5-5)	5 (5-5)	5 (5-5)	5 (5-5)	5 (5-5)	5 (5-5)	5 (5-5)	5 (5-5)	5 (5-5)	5 (5-5)	5 (5-5)
Root Canals	5 (5-5)	5 (5-5)	5 (5-5)	5 (5-5)	5 (5-5)	5 (5-5)	5 (5-5)	5 (5-5)	5 (5-5)	5 (5-5)	5 (5-5)	5 (5-5)	5 (5-5)
Apical Foramen	5 (5-5)	5 (5-5)	5 (5-5)	5 (5-5)	5 (5-5)	5 (5-5)	5 (5-5)	5 (5-5)	5 (5-5)	5 (5-5)	5 (5-5)	5 (5-5)	5 (5-5)

**Fig. 6:**
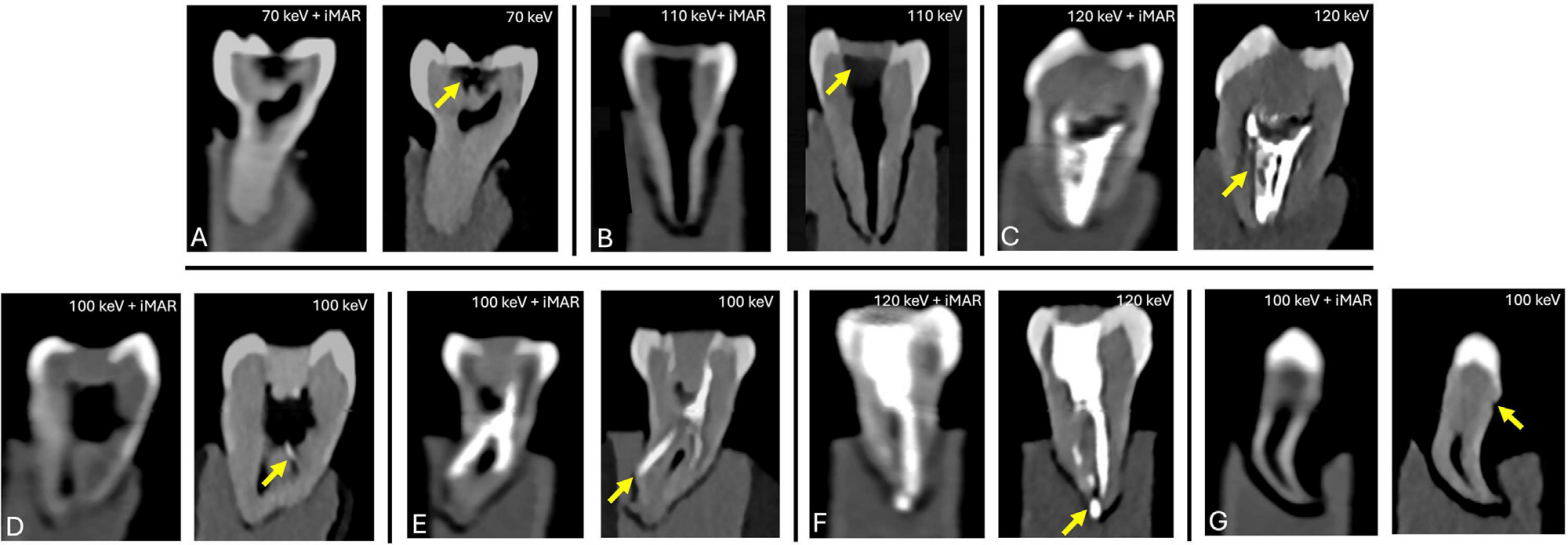
Reconstructions with iMAR compared to corresponding optimal VMI levels without iMAR for the following pathologies: (A) Caries; (B) Instrumented root canal with Cavit; (C) Gutta-percha obturation with sealer; (D) Fractured endodontic Hedstrom file; (E) Root perforation; (F) Post placement with root perforation; and (G) External root resorption.

Reconstructions using iMAR consistently resulted in reduced depiction quality, ranging from non-diagnostic (fractured endodontic files, finest external resorption) to good (caries, instrumented canals with Cavit), without any improvement in artifact reduction compared to the corresponding VMI levels without iMAR. Comparison between the task-specific optimal VMI reconstructions with and without iMAR using the Wilcoxon signed-rank test revealed no significant differences (*p* > .05), indicating no measurable benefit of iMAR (Figure 6, Table 4).

**Table 4:** Task-specific analysis of depiction quality and artifact susceptibility at the task-specific optimal VMI level with iMAR, evaluated across various endodontic diagnostic tasks and associated complications

	Imaging protocol	Depiction quality of endodontic challenge	Artifacts
Caries	***70keV + iMAR***	4 (4-4)	4 (4-4)
Instrumented root canal with Cavit	***110keV + iMAR***	4 (4-4)	5 (5-5)
Gutta percha obturation with sealer	***120keV + iMAR***	3 (3-3)	2 (2-2.75)
Fractured endodontic file	***100keV + iMAR***	1 (1-1)	4 (4-4)
Root perforation	***100keV + iMAR***	3.5 (3-4)	4 (4-4)
Post placement with root perforation	***120keV + iMAR***	3 (2-4)	2 (2-2)
External root resorption	***100keV + iMAR***	2 (2-2)	4 (4-4)

## Discussion

This *ex vivo* feasibility study demonstrated that VMI from PCD-CT offer high diagnostic accuracy and excellent depiction quality, with minimal artifacts across the evaluated VMI range (70-190 keV) for a variety of simulated endodontic lesions and complications. Optimal visualization of key endodontic anatomical structures and complex pathologies was achieved with VMI at 100 keV or higher. In contrast, VMI at lower energy levels (70-100 keV) enhanced the depiction of low-density features, such as carious lesions and fractured instruments, although with a slight increase in artifacts. Dedicated metal artifact reduction algorithms did not further improve image quality or reduce artifacts compared to the corresponding VMI levels without iMAR.

Previous studies compared polychromatic PCD-CT and CBCT across varying radiation dose levels for dental anatomy, apical osteolysis, and both quantitative and qualitative assessments of sequestra, fractures, cystic lesions, and extended periodontal gaps.^12^^,^^20^ PCD-CT demonstrated superior image quality, significantly reduced artifacts, and up to 37% higher contrast-to-noise ratios (CNRs), enabling better tissue contrast differentiation at one-fourth the radiation dose of CBCT.^20^ In terms of detection, PCD-CT outperformed CBCT, especially at lower doses, with excellent inter-reader agreements (κ = 0.744-1) and highly precise quantitative measurements, confirming clinical reliability.^21^ Vanden Broeke et al. validated the feasibility of visualizing fine endodontic anatomy with a resolution comparable to that of CBCT, while simultaneously providing superior metal artifact reduction.^22^ In the context of endodontic patho-anatomical challenges, Fontenele et al.^11^ compared PCD-CT with multiple CBCT systems for visualizing fine endodontic structures (apical delta, narrow canals, isthmuses) and root cracks. Both modalities demonstrated comparable performance in detecting fine anatomical features, except for root cracks, where high-resolution CBCT outperformed PCD-CT.^11^

To our knowledge, this is the first study to assess PCD-CT-based VMIs for endodontic diagnostics. The obtained results expand the existing evidence in head and neck applications of PCD-CT and highlight the potential of spectral imaging techniques to improve image quality, reduce beam-hardening artifacts, and enhance contrast resolution,^23^^,^^24^ as evidenced by the consistently high depiction scores and the absence or minimal presence of artifacts in this study. The trends identified in this study may be attributed to the interaction between tissue composition and energy-dependent imaging characteristics. Carious lesions were consistently depicted with high diagnostic confidence and detailed patho-anatomical delineation on VMI at lower energies, primarily due to the pronounced contrast between demineralized and the healthy surrounding tooth structure. Using VMI at higher energy levels, the predominance of Compton scattering results in a progressive reduction of tissue contrast, thereby diminishing the visibility of morphological details within carious lesions. Regarding filling materials, Cavit exhibited no noticeable influence on image depiction across all VMI levels. In contrast, gutta-percha combined with sealer revealed structural details at the highest VMI energies that cannot be visualized by CBCT, enabling the differentiation between the gutta-percha core and the surrounding sealer (Figure 5). Such fine detail may offer novel insights into the causes of endodontic therapy failures by allowing precise assessment of the filling integrity and material interfaces. Fractured endodontic files were consistently identified within the optimal VMI range of 70-100 keV, where a favorable balance between artifact suppression and structural delineation was achieved for the examined Hedstrom files. Although minor artifacts were present at lower keV levels, they did not compromise diagnostic interpretability, allowing for reliable detection of metallic fragments.

The application of iMAR in combination with task-oriented VMI did not provide superior artifact suppression or improved depiction quality. This observation is most likely related to the technical constraint that iMAR can only be applied with reconstruction kernels up to a sharpness level of Hr56. Consequently, these reconstructions yielded reduced diagnostic detail across all assessed pathologies and rendered fine external resorptions or metallic structures, such as fractured endodontic files, partially or entirely undetectable. Therefore, our findings suggest that, for high-quality endodontic imaging, the combination of VMI at high energy levels and a sharp reconstruction kernel remains the most effective strategy. It should be noted that, in the absence of iMAR, a sharper kernel was deliberately selected in order to fully leverage the resolution capabilities of PCD-CT.

Furthermore, recent advances in dental imaging have introduced quantitative image analysis approaches, such as trabecular bone assessment using CBCT to predict surgical outcomes.^25^^,^^26^ Future investigations may incorporate similar quantitative methodologies to complement the present findings and further characterize the technical impact of spectral reconstruction and artifact-reduction algorithms. However, the applicability and value of such quantitative approaches for spectral PCD-CT–based endodontic imaging need to be assessed.

The strength of this study lies in its comprehensive, task-specific assessment across a broad spectrum of clinically relevant endodontic scenarios, supported by thorough observer calibration and blinded assessments, independent of the observer’s experience and subspecialization. Additionally, the use of a standardized dose-matching approach ensures a fair comparison with the established CBCT reference standard, highlighting the potential of PCD-CT to deliver superior image quality without increased radiation exposure.

However, this feasibility study has several limitations. First, the findings do not fully replicate the clinical environment, where factors such as patient movement, soft tissue attenuation, and biological variability could affect imaging performance. Second, the small sample size limits the generalizability of the results. Third, objective parameters such as signal-to-noise ratio (SNR) and CNR were not included in the present analysis, as such measurements not necessarily translatable to clinically meaningful improvements in image quality.^27^, ^28^, ^29^ Additionally, the heterogeneity of simulated endodontic tasks and material compositions precluded uniform quantitative artifact assessment across all specimens. Fourth, the high costs and limited availability of PCD-CT are still considered a challenge regarding its adaptation for dentomaxillofacial imaging. Thus, future studies should aim to validate these findings in larger clinical cohorts, potentially exploring dose reduction strategies and task-based VMI level selection tailored to comprehensive endodontic therapy.

## Conclusion

VMI from PCD-CT provides high quality imaging with minimal artifacts, well-suited for indication-specific endodontic diagnostics. While the routine application of iMAR did not offer additional benefits in this context, further research is required to explore the full clinical potential and optimize imaging protocols for daily clinical use.

## Author contributions

Conceptualization, design, execution, or analysis, A.A.H., S.D., V.M., T.T.D., H.A., T.F., H.E., B.S., E.B.; drafting manuscript, A.A.H. and S.D., writing review and editing, V.M., T.T.D., H.A., T.F., H.E., B.S., E.B. All authors have read and agreed to the final version of the manuscript. All authors agreed to be accountable for all aspects of the work.

## Conflict of interest

The authors declare the following financial interests/personal relationships which may be considered as potential competing interests: Victor Mergen, Hatem Alkadhi, Tristan Demmert, Egon Burian, Thomas Flohr reports a relationship with Bayer, Canon, Guerbet, and Siemens. that includes: funding grants. If there are other authors, they declare that they have no known competing financial interests or personal relationships that could have appeared to influence the work reported in this paper.
